# Caffeine Consumption and Mortality in Diabetes: An Analysis of NHANES 1999–2010

**DOI:** 10.3389/fendo.2018.00547

**Published:** 2018-09-20

**Authors:** João Sérgio Neves, Lia Leitão, Rita Magriço, Miguel Bigotte Vieira, Catarina Viegas Dias, Ana Oliveira, Davide Carvalho, Brian Claggett

**Affiliations:** ^1^Department of Endocrinology, Diabetes and Metabolism, São João Hospital Center, Porto, Portugal; ^2^Department of Surgery and Physiology, Faculty of Medicine, Cardiovascular Research Center, University of Porto, Porto, Portugal; ^3^Neurology Department, Hospital Prof. Doutor Fernando Fonseca, Amadora, Portugal; ^4^Nephrology Department, Hospital Curry Cabral, Lisbon, Portugal; ^5^Nephrology and Renal Transplantation Department, Centro Hospitalar Lisboa Norte, Lisbon, Portugal; ^6^NOVA Medical School, Lisbon, Portugal; ^7^Faculty of Medicine, Instituto de Investigação e Inovação em Saúde, University of Porto, Porto, Portugal; ^8^Cardiovascular Division, Brigham and Women's Hospital, Harvard Medical School, Boston, MA, United States

**Keywords:** caffeine, coffee, mortality, diabetes, national health and nutrition examination survey

## Abstract

**Aim:** An inverse relationship between coffee consumption and mortality has been reported in the general population. However, the effect of coffee consumption in diabetes remains unclear. We aimed to evaluate the association of caffeine consumption and caffeine source with mortality among patients with diabetes.

**Methods:** We examined the association of caffeine consumption with mortality among 1974 women and 1974 men with diabetes, using the National Health and Nutrition Examination Survey (NHANES) 1999–2010. Caffeine consumption was assessed at baseline using 24 h dietary recalls. Cox proportional hazard models were fitted to estimate hazard ratios (HR) for all-cause, cardiovascular, and cancer-related mortality according to caffeine consumption and its source, adjusting for potential confounders.

**Results:** A dose-dependent inverse association between caffeine and all-cause mortality was observed in women with diabetes. Adjusted HR for death among women who consumed caffeine, as compared with non-consumers, were: 0.57 (95% CI, 0.40–0.82) for <100 mg of caffeine/day, 0.50 (95% CI, 0.32–0.78) for 100 to <200 mg of caffeine/day, and 0.39 (95% CI, 0.23–0.64) for ≥200 mg of caffeine/day (*p* = 0.005 for trend). This association was not observed in men. There was a significant interaction between sex and caffeine consumption (*p* = 0.015). No significant association between total caffeine consumption and cardiovascular or cancer mortality was observed. Women who consumed more caffeine from coffee had reduced risk of all-cause mortality (*p* = 0.004 for trend).

**Conclusion:** Our study showed a dose-dependent protective effect of caffeine consumption on mortality among women with diabetes.

## Introduction

Diabetes is a major public health problem with an increasing prevalence worldwide (1). Given its significant burden, it is important to identify lifestyle factors for improvement of prognosis. Caffeine is provided in different sources, mainly coffee, tea, and soft drinks. Coffee contains more caffeine than the majority of foods and constitutes one of the most commonly consumed beverages worldwide. The detection of a health-related effect associated with coffee consumption may have a potential great impact in public health (2). Coffee has been referred as containing several bioactive compounds including antioxidants with potentially beneficial properties. An inverse association between coffee consumption and serum biomarkers of inflammation and insulin resistance has been described (3–5). A meta-analysis of prospective studies (6) and a systematic review (7) showed that coffee consumption might be associated with reduction in the incidence of type 2 diabetes.

A recent dose-response systematic review concluded that coffee consumption was strongly associated with a risk reduction in all-cause and cardiovascular disease (CVD) mortality. However, this systematic review excluded studies which analyzed specific subpopulations, such as those including only people with diabetes (8). Although caffeine consumption appears to be associated with a decreased risk of developing type 2 diabetes, it is unclear if its protective effect persists in people with established diabetes. In a prospective study including 4,365 patients with a prior myocardial infarction, drinking coffee was associated with lower risk of cardiovascular mortality and ischemic heart disease mortality (9). In this study, significant inverse associations were reported in patients without diabetes, whereas the associations were weak and non-significant in the smaller group with diabetes.

After reviewing the literature, we found only three studies specific of patients with diabetes. One prospective cohort of 7,170 female registered nurses with diabetes found that habitual coffee consumption was not associated with increased risk for cardiovascular diseases or premature mortality (10). A similar cohort of 3,497 male health professionals with diabetes found no association between caffeine consumption and CVD or all-cause mortality (11). Another study, restricted to a Finnish population (3,837 patients), found that in patients with type 2 diabetes, coffee drinking was associated with reduced all-cause, CVD, and coronary heart disease mortality (12). However, in this study, no adjustments were made for diabetes duration, complications of diabetes, and insulin treatment.

Considering that there is limited and conflicting evidence regarding the relationship between caffeine consumption and mortality in people with diabetes, we examined the continuous National Health and Nutrition Examination Survey (NHANES) 1999–2010 database to evaluate the effect of caffeine consumption and caffeine source on all-cause, cardiovascular, and cancer mortality among patients with diabetes.

## Materials and methods

### Study design and participants

We performed an analysis of the continuous NHANES database. The NHANES is a periodic survey conducted by the National Center for Health Statistics (NCHS) of the Centers for Disease Control and Prevention (CDC). NHANES is a stratified, multi-stage survey using a nationally representative sample of the non-institutionalized civilian population of the United States. Participants are selected at random through a complex statistical process each year, and they complete personal structured interviews at home and then perform a physical examination at a mobile examination center that includes height, weight, and laboratory measurements (13). We used data from 1999 to 2010, that includes 62,160 people. We restricted our analysis to individuals with ≥18-year-old (35,379 subjects) and with diabetes (4,544 subjects). Diabetes was defined by a self-reported previous diagnosis, a hemoglobin A1c level of ≥6.5%, or a fasting plasma glucose level of ≥126 mg/dL. Both patients with type 1 and type 2 diabetes were included. We excluded 596 subjects due to implausible alimentary reports (as defined in previous studies: consumption of <500 kcal/day or >3500 kcal/day) (14) or missing information on caffeine consumption and/or mortality. Finally, 3,948 subjects were included in our present analysis. The NCHS Research Ethics Review Board reviewed and approved NHANES, and all participants provided written informed consent. This study was registered at www.clinicaltrials.gov as NCT03367806.

### Assessment of exposure

In all cycles of NHANES 1999–2010, a 24-h dietary recall was collected. Using an automated multiple-pass method, all food items and quantities consumed in the 24 h preceding the interview were recorded. For participants in the 1999–2002 NHANES, only one in-person 24-h dietary recall was administered. The cycles starting from 2003 onward included two recalls, the first one in-person and the second one via telephone collected 3 to 10 days following the first dietary interview but not on the same day of the week. To calculate the caffeine, energy, and nutrient intakes, for participants in the 1999–2002 NHANES, we used the nutritional information from foods and beverages collected in the single 24-h dietary recall. For participants in the 2003-2010 NHANES, the mean of the nutritional information from both recalls was used (15). NHANES includes information regarding nutrient source by type of food ingested. This data was used to ascertain the quantity of ingested caffeine originating in coffee, tea, or soft drink for each patient. The impact of caffeine consumption obtained from each of the three types of drink on different outcomes was evaluated.

Considering the mean caffeine content per unit of caffeinated beverage (95 mg in 8 oz. of coffee, 48 mg in 8 oz. of tea, and 30 mg in 12 oz. of cola) (16), we divided the daily intake of caffeine from all sources and from coffee into three categories (<100 mg, 100 to <200 mg, and 200 mg or more). Given the low number of patients with caffeine intake from tea and from soft drinks, and the high variability of caffeine in these beverages, those patients were divided into tertiles of consumption and are presented as Supplementary Material.

Continuous variables are presented as means with standard deviations except for polyunsaturated to saturated fatty acids ratio, fibers per day, and years of diabetes which were summarized using median (interquartile range) due to their right-skewed distributions. Categorical variables are presented as percent with 95% confidence intervals.

### Outcomes

The primary outcome was time to death. As secondary outcomes, we selected time to cardiovascular death and time to death by cancer. Mortality status and cause of death were determined by NHANES linked National Death Index public-access files through December 31, 2011.

### Statistical analysis

All calculations took into account the complex survey design of the NHANES dataset and were analyzed according to the CDC analytic recommendations (17).

To assess the crude association between caffeine consumption and time to death, we performed a Kaplan-Meier curve and log-rank test. We performed further analysis using the Cox proportional hazards models to adjust for potential confounders. We built two different Cox Proportional Hazard models to analyze the primary outcome: one model including age at baseline, race (Mexican American, other Hispanic, non-Hispanic white, non-Hispanic black, other race), annual family income (<$25000, $25000 to $75000, >$75000), smoking status (never smoker, current smoker, or former smoker), and diabetic nephropathy (glomerular filtration rate <60 mL/min/1.73 m^2^ or urine albumin/creatinine ratio ≥300 mg/g) (model 1); Model 2 including all model 1 covariates plus body mass index (BMI) (<20.0, 20.0 to <25.0, 25.0 to <30.0, 30.0 to <35.0, 35.0 to <40.0, ≥40.0 kg/m2), education level [less than 9th grade, 9-11th grade, high-school grade, some college or associate's (AA) degree, college graduate and above], daily carbohydrate consumption (grams of carbohydrate per 100 kcal), alcohol consumption (no alcohol consumption, <20 grams/day, ≥20 grams/day), years since diabetes diagnosis (undiagnosed, ≤5 years, 5 to ≤15 years, >15 years), diagnosis of hypertension, retinopathy, macrovascular complications (coronary artery disease, history of myocardial infarction, or history of stroke), insulin treatment and survey cycle (years 1999–2000, 2001–2002, 2003–2004, 2005–2006, 2007–2008, or 2009–2010). Regarding cause-specific mortality, we only used the more restrictive model (model 1), due to the low number of outcome events. We tested for interactions between caffeine consumption and the other 14 variables in model 2 for all-cause mortality.

We also considered physical activity as an important potential confounder. Physical activity was measured differently along the various NHANES cycles. Therefore, we chose to use variables that allowed categorization of physical activity level into three categories (low, intermediate, and high), to combine them into a single variable. From 1999 to 2006 the physical activity level was assessed with the question “compare activity with others of the same age” (participants were classified into approximate tertiles as low if “less active,” as intermediate if “about the same,” and as high if “more active,” with 31, 28, and 41% of participants, respectively, falling into these categories). From 2007 to 2010 the weekly metabolic equivalents (MET) minutes of physical activity (accounting for vigorous work-related activity, moderate work-related activity, walking or bicycling for transportation, vigorous leisure-time physical activity, and moderate leisure-time physical activity) was divided into tertiles (participants were classified as low if included in the lower MET-minute tertile, as intermediate if in the middle MET-minute tertile, and as high if in the higher MET-minute tertile). The results of mortality associated with caffeine consumption adjusted for physical activity are presented in the Supplementary Material.

As sensitivity analysis, we additionally performed the Cox proportional hazards models using daily intake of caffeine (from all sources and from coffee, tea, or soft drinks) as a continuous variable.

Multiple imputation by chained equations was used for dealing with missing data regarding covariates. Twenty imputations per missing observation were performed and analyzed. A test for trend over increasing caffeine consumption categories was performed where each category median was modeled as a continuous variable in the regression. A two-sided *p*-value of <0.05 was considered statistically significant. Analyses were performed with Stata (version 14.2).

## Results

### Association of caffeine consumption with dietary and lifestyle factors

Caffeine consumption at baseline was associated with several other dietary and lifestyle factors, with some differences according to sex (Table 1). In both men and women, compared with people who did not drink caffeine-containing beverages, caffeine consumers were more likely to be non-Hispanic white and current smokers, to have a higher level of education, to have an annual family income higher than $25000, and to consume less carbohydrates and less fibers per kilocalorie ingested. Women that consumed caffeine were more likely to be treated with insulin, had less diabetic kidney disease, and a lower ratio of polyunsaturated/saturated fatty acids intake.

**Table 1:** Baseline characteristics of the study population.

**Baseline characteristics according to caffeine consumption among women (*****n*** = **1974)**								
	**No consumption (*****n*** = **219)**	<**100 mg/day (*****n*** = **979)**	***P-***value^*^	**100 to**<**200 mg/day (*****n*** = **438)**	***P-***value^*^	≥**200 mg/day (*****n*** = **338)**	***P-***value^*^	**P for trend**
Age, years	58.3 (15.8)	61.6 (15.0)	0.022	59.5 (14.3)	0.469	58.6 (13.2)	0.829	**0.041**
Non-Hispanic White, %	34.7%	53.0%	0.001	59.2%	<0.001	79.2%	<0.001	<**0.001**
Annual family income <$25000, %	51.4%	48.8%	0.399	39.1%	0.018	38.6%	0.010	**0.004**
Education level -Less than 9th grade, %	22.0%	15.9%	0.119	10.1%	0.002	9.1%	<0.001	<**0.001**
Current smokers, %	6.3%	8.4%	0.312	12.7%	0.029	25.9%	<0.001	<**0.001**
Former smokers, %	24.0%	26.5%	0.548	34.3%	0.053	33.2%	0.079	**0.023**
Alcohol consumption >20 grams/day, %	2.3%	2.9%	0.651	5.2%	0.172	4.9%	0.219	0.126
Carbohydrates per day, gram/100 kcal	12.9 (3.0)	12.7 (2.3)	0.448	12.1 (2.3)	0.020	11.8 (2.5)	0.001	<**0.001**
Polyunsaturated/saturated fatty acids ratio	0.73 (0.52–1.11)	0.70 (0.50–0.94)	0.061	0.67 (0.49–0.92)	0.056	0.65 (0.49–0.84)	0.001	**0.001**
Fiber per day, gram/100 kcal	0.95 (0.54–1.27)	0.91 (0.65–1.16)	0.957	0.85 (0.64–1.09)	0.107	0.78 (0.59–1.03)	0.045	**0.002**
Low physical activity level, %	44.9%	37.2%	0.109	37.4%	0.201	41.9%	0.607	0.469
BMI, kg/m2	34.7 (8.9)	33.2 (8.3)	0.083	33.0 (6.9)	0.034	33.8 (7.8)	0.342	0.775
Hypertension, %	67.3%	67.2%	0.978	72.5%	0.266	67.0%	0.957	0.889
Dyslipidemia, %	53.1%	60.3%	0.188	61.3%	0.128	58.7%	0.283	0.926
Time since diagnosis of diabetes, years	3 (0–13)	5 (0–12)	0.100	5 (1–13)	0.415	7 (1–15)	0.092	0.592
Diabetic retinopathy, %	24.0%	21.7%	0.591	22.0%	0.722	25.4%	0.661	0.219
Diabetic kidney disease, %	23.2%	26.4%	0.511	27.8%	0.477	16.8%	0.100	**0.005**
Macrovascular complications, %	19.3%	18.1%	0.696	19.2%	0.988	19.5%	0.908	0.629
Insulin treatment, %	21.5%	18.6%	0.465	21.0%	0.876	27.6%	0.274	**0.021**
**Baseline characteristics according to caffeine consumption among men (*****n*** = **1974)**								
	**No consumption (*****n*** = **186)**	<**100 mg/day (*****n*** = **739)**	***P***-value^*^	**100 to**<**200 mg/day (*****n*** = **470)**	***P***-value^*^	≥**200 mg/day (*****n*** = **579)**	***P***-value^*^	**P for trend**
Age, years	57.3 (14.6)	60.0 (14.0)	0.080	58.0 (14.0)	0.660	58.1 (12.4)	0.601	0.245
Non-Hispanic White, %	41.4%	54.3%	0.013	70.4%	<0.001	80.3%	<0.001	<**0.001**
Annual family income <$25000, %	35.2%	34.7%	0.871	27.6%	0.110	27.9%	0.123	**0.029**
Education level - Less than 9th grade, %	18.2%	16.3%	0.616	12.6%	0.126	10.2%	0.020	**0.001**
Current smokers, %	16.0%	11.8%	0.300	14.8%	0.826	25.7%	0.097	<**0.001**
Former smokers, %	39.7%	45.4%	0.247	46.7%	0.199	45.1%	0.366	0.834
Alcohol consumption >20 grams/day, %	15.9%	9.8%	0.073	11.4%	0.302	11.6%	0.169	0.867
Carbohydrates per day, gram/100 kcal	11.9 (3.1)	11.8 (2.5)	0.958	11.7 (2.4)	0.573	11.3 (2.6)	0.124	**0.006**
Polyunsaturated/saturated fatty acids ratio	0.70 (0.47–0.98)	0.68 (0.48–0.91)	0.306	0.70 (0.50–0.91)	0.249	0.64 (0.47–0.88)	0.133	0.216
Fiber per day, gram/100 kcal	0.83 (0.55–1.10)	0.83 (0.61–1.14)	0.624	0.76 (0.55–1.09)	0.444	0.78 (0.57–0.99)	0.134	**0.010**
Low physical activity level, %	40.9%	30.0%	0.030	32.8%	0.157	33.4%	0.120	0.773
BMI, kg/m^2^	31.9 (8.1)	31.2 (6.1)	0.481	31.4 (6.0)	0.601	32.4 (7.3)	0.661	0.102
Hypertension, %	66.7%	60.5%	0.239	60.1%	0.278	55.1%	0.046	0.062
Dyslipidemia, %	55.6%	58.2%	0.666	60.0%	0.486	63.0%	0.207	0.166
Time since diagnosis of diabetes, years	3 (0–11)	4 (0–11)	0.882	3 (0–10)	0.539	5 (0–12)	0.966	0.779
Diabetic retinopathy, %	26.4%	26.1%	0.932	18.3%	0.223	18.6%	0.432	0.198
Diabetic kidney disease, %	15.1%	25.0%	0.010	16.9%	0.639	20.1%	0.197	0.408
Macrovascular complications, %	23.2%	24.8%	0.662	20.5%	0.551	26.2%	0.448	0.378
Insulin treatment, %	20.9%	21.8%	0.814	21.6%	0.888	23.2%	0.614	0.563

*Baseline population characteristics according to caffeine consumption among women and men*.

* *vs no consumption group. Kcal, kilocalorie; BMI, Body mass index. Significant P for trend values are shown in bold*.

### Caffeine consumption and mortality

**Figure 1:**
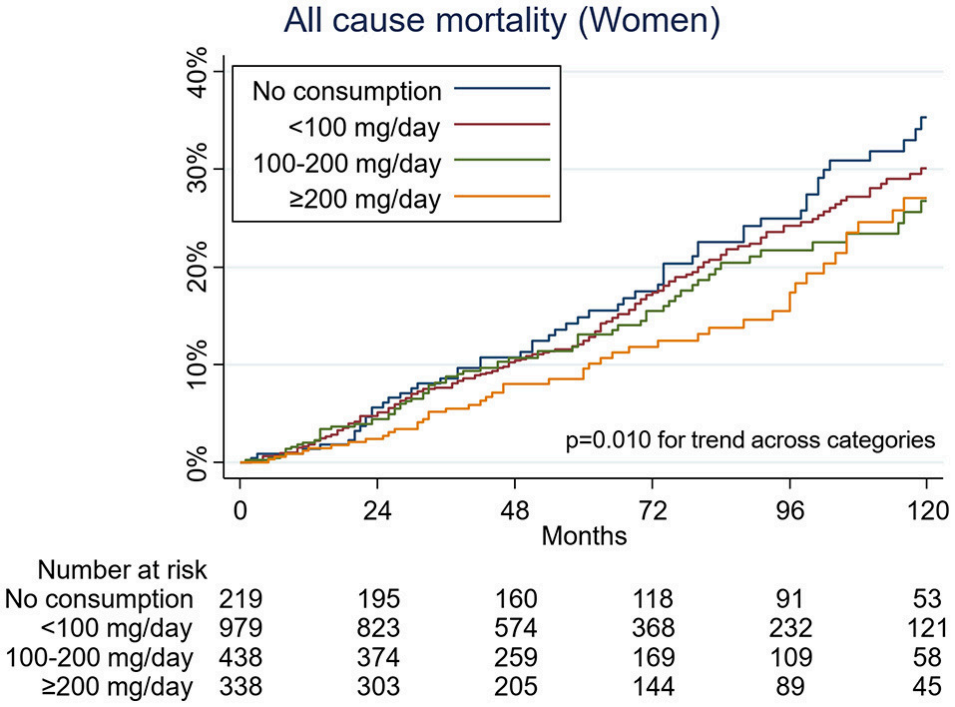
Kaplan-Meier curves for all-cause mortality by caffeine consumption among women.

**Figure 2:**
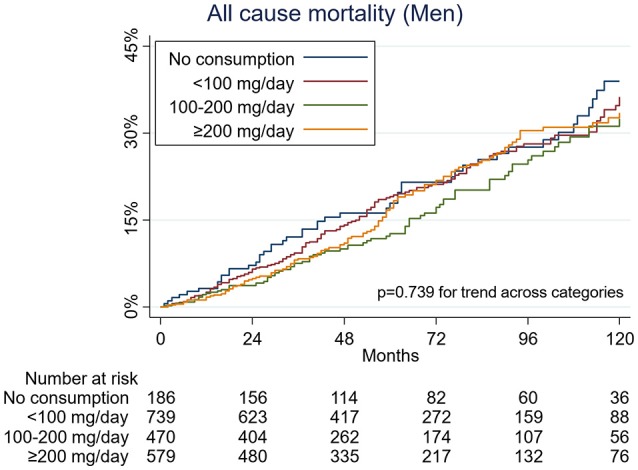
Kaplan-Meier curves for all-cause mortality by caffeine consumption among men.

During a median 57 months of follow-up (total person-years, 21,606), 407 men and 351 women died. There was a significant interaction between sex and caffeine consumption with respect to mortality (*p* = 0.015 for interaction in model 2). In the unadjusted analysis (Figure 1), and also after multivariate analysis, caffeine consumption was associated with a decrease in all-cause mortality in women (*p* = 0.005 for trend across categories, in model 2). A dose-dependent inverse association between caffeine and all-cause mortality was observed. Hazard ratios (HR) for death among women who consumed caffeine, as compared with women who did not consume caffeine, were as follows: 0.57 (95% confidence interval [CI], 0.40 to 0.82) for less than 100 mg of caffeine per day, 0.50 (95% CI, 0.32 to 0.78) for 100 to less than 200 mg of caffeine, and 0.39 (95% CI, 0.23 to 0.64) for 200 mg or more of caffeine per day (Table 2). In contrast, this association was not observed among men (Figure 2), even after adjustment for potential confounders (Table 2).

**Table 2:** Association of caffeine consumption with mortality.

**Association of caffeine consumption with mortality among women**						
	**No consumption (*****n*** = **219)**	<**100 mg/day (*****n*** = **979)**	**100 to**<**200 mg/day (*****n*** = **438)**	≥**200 mg/day (*****n*** = **338)**	**P for trend**	**Continuous analysis**^a^
**All-cause mortality**						
No. of deaths (%)	59 (26.9%)	170 (17.4%)	73 (16.7%)	49 (14.5%)		351 (17.8%)
Unadjusted HR	–	0.71 (0.51–1.00)	0.63 (0.42–0.96)	0.46 (0.27–0.78)	**0.010**	**0.86 (0.76**–**0.96)**
Model 1 HR	–	0.60 (0.43–0.83)	0.52 (0.34–0.80)	0.42 (0.25–0.71)	**0.020**	**0.88 (0.77**–**0.99)**
Model 2 HR	–	0.57 (0.40–0.82)	0.50 (0.32–0.78)	0.39 (0.23–0.64)	**0.005**	**0.86 (0.76**–**0.96)**
**CVD mortality**						
No. of deaths (%)	16 (7.3%)	40 (4.1%)	15 (3.4%)	8 (2.4%)		79 (4.0%)
Unadjusted HR	–	0.66 (0.33–1.30)	0.45 (0.22–0.93)	0.40 (0.15–1.08)	0.118	**0.82 (0.68**–**0.99)**
Model 1 HR	–	0.52 (0.26–1.02)	0.37 (0.18–0.76)	0.38 (0.14–1.00)	0.208	0.86 (0.70–1.06)
**Cancer mortality**						
No. of deaths (%)	9 (4.1%)	26 (2.7%)	11 (2.5%)	8 (2.4%)		54 (2.7%)
Unadjusted HR	–	0.87 (0.34–2.22)	0.70 (0.24–2.02)	0.49 (0.13–1.80)	0.204	0.88 (0.65–1.19)
Model 1 HR	–	0.71 (0.27–1.89)	0.52 (0.17–1.59)	0.35 (0.08–1.53)	0.126	0.84 (0.59–1.20)
**Association of caffeine consumption with mortality among men**						
	**No consumption (*****n*** = **186)**	<**100 mg/day (** = **739)**	**100 to**<**200 mg/day (*****n*** = **470)**	≥**200 mg/day (*****n*** = **579)**	**P for trend**	**Continuous analysis**^a^
**All-cause mortality**						
No. of deaths (%)	50 (26.9%)	159 (21.5%)	82 (17.5%)	116 (20.0%)		407 (20.6%)
Unadjusted HR	–	1.24 (0.81–1.91)	1.00 (0.64–1.57)	1.21 (0.81–1.82)	0.739	1.03 (0.97–1.09)
Model 1 HR	–	0.94 (0.62–1.41)	0.77 (0.49–1.23)	0.91 (0.60–1.39)	0.925	1.04 (0.98–1.10)
Model 2 HR	–	1.09 (0.70–1.69)	0.90 (0.55–1.47)	1.01 (0.65–1.56)	0.783	1.03 (0.97–1.10)
**CVD mortality**						
No. of deaths (%)	11 (5.9%)	50 (6.8%)	23 (4.9%)	36 (6.2%)		120 (6.1%)
Unadjusted HR	–	2.09 (0.99–4.39)	1.59 (0.58–4.33)	1.92 (0.85–4.31)	0.749	1.07 (0.98–1.18)
Model 1 HR	–	1.47 (0.70–3.12)	1.13 (0.41–3.10)	1.18 (0.54–2.58)	0.517	1.07 (0.98–1.16)
**Cancer mortality**						
No. of deaths (%)	12 (6.5%)	28 (3.8%)	18 (3.8%)	21 (3.6%)		79 (4.0%)
Unadjusted HR	–	1.15 (0.51–2.63)	0.96 (0.37–2.47)	1.17 (0.52–2.62)	0.830	1.09 (0.97–1.22)
Model 1 HR	–	1.03 (0.42–2.54)	0.78 (0.28–2.13)	0.90 (0.37–2.18)	0.777	1.07 (0.95–1.22)

*Model 1: Adjusted for age, race, annual family income, smoking status, and diabetic kidney disease. Model 2: Adjusted for covariates in Model 1 and body mass index, education level, daily carbohydrate consumption, alcohol consumption, years since diabetes diagnosis, diagnosis of hypertension, retinopathy, macrovascular complications, insulin treatment and survey cycle*.

a *HR for the continuous analysis are presented for each 100 mg increase in caffeine consumption. HR, Hazard Ratio; CVD, Cardiovascular disease. Significant P for trend values and significant hazard ratios in the continuous analysis are shown in bold*.

Specific causes of death were also examined. There were 199 deaths from CVD and 133 deaths due to cancer during the follow-up. After multivariate adjustment, there was no significant association between caffeine consumption and deaths from CVD or cancer, both in men and women (Table 2).

### Source of caffeine consumption and mortality

An analysis of caffeine consumption according to its origin on coffee, tea, or soft drinks was also performed. In the unadjusted analysis, and also after multivariate analysis, a reduced risk in all-cause mortality was observed in women with diabetes who consumed caffeine from coffee (Table 3). The adjusted hazard ratios were as follows: 0.74 (95% CI, 0.53 to 1.02) for less than 100 mg of caffeine per day, 0.71 (95% CI, 0.46 to 1.09) for 100 mg to less than 200 mg of caffeine, and 0.53 (95% CI, 0.35 to 0.80) for 200 mg or more of caffeine per day (*p* = 0.004 for trend across categories, in model 2). There were no significant associations of caffeine consumption from coffee with cardiovascular or cancer mortality.

**Table 3:** Association of caffeine consumption from coffee with mortality.

**Association of caffeine consumption from coffee with mortality among women**						
	**No consumption (*****n*** = **734)**	<**100 mg/day (*****n*** = **707)**	**100 to**<**200 mg/day (*****n*** = **316)**	≥**200 mg/day (*****n*** = **217)**	**P for trend**	**Continuous analysis**^a^
**All-cause mortality**						
No. of deaths (%)	127 (17.3%)	130 (18.4%)	58 (18.4%)	36 (16.6%)		351 (17.8%)
Unadjusted HR	–	1.25 (0.94–1.66)	1.09 (0.75–1.60)	0.73 (0.50–1.09)	0.077	**0.91 (0.83**–**0.99)**
Model 1 HR	–	0.79 (0.60–1.04)	0.74 (0.47–1.15)	0.57 (0.38–0.85)	**0.009**	**0.87 (0.78**–**0.98)**
Model 2 HR	–	0.74 (0.53–1.02)	0.71 (0.46–1.09)	0.53 (0.35–0.80)	**0.004**	**0.85 (0.76**–**0.95)**
**CVD mortality**						
No. of deaths (%)	31 (4.2%)	32 (4.5%)	11 (3.5%)	5 (2.3%)		79 (4.0%)
Unadjusted HR	–	1.51 (0.86–2.66)	0.98 (0.42–2.26)	0.50 (0.18–1.41)	0.123	**0.83 (0.69**–**0.99)**
Model 1 HR	–	0.96 (0.56–1.66)	0.72 (0.28–1.85)	0.42 (0.14–1.25)	0.110	0.81 (0.64–1.02)
**Cancer mortality**						
No. of deaths (%)	19 (2.6%)	18 (2.6%)	12 (3.8%)	5 (2.3%)		54 (2.7%)
Unadjusted HR	–	1.66 (0.80–3.42)	1.81 (0.74–4.38)	0.89 (0.23–3.41)	0.905	0.99 (0.76–1.29)
Model 1 HR	–	1.16 (0.54–2.48)	1.24 (0.47–3.23)	0.59 (0.15–2.39)	0.392	0.91 (0.64–1.29)
**Association of caffeine consumption from coffee with mortality among men**						
	**No consumption (*****n*** = **656)**	<**100 mg/day (*****n*** = **572)**	**100 to**<**200 mg/day (*****n*** = **358)**	≥**200 mg/day (*****n*** = **388)**	**P for trend**	**Continuous analysis**^a^
**All-cause mortality**						
No. of deaths (%)	132 (20.1%)	126 (22.0%)	70 (19.6%)	79 (20.4%)		407 (20.6%)
Unadjusted HR	–	1.59 (1.19–2.13)	1.13 (0.77–1.66)	1.42 (0.93–2.08)	0.282	1.06 (1.00–1.12)
Model 1 HR	–	1.05 (0.76–1.45)	0.80 (0.57–1.11)	1.07 (0.74–1.55)	0.922	1.03 (0.96–1.11)
Model 2 HR	–	1.04 (0.74–1.47)	0.76 (0.53–1.08)	1.09 (0.76–1.56)	0.873	1.03 (0.96–1.11)
**CVD mortality**						
No. of deaths (%)	34 (5.2%)	45 (7.9%)	18 (5.0%)	23 (5.9%)		120 (6.1%)
Unadjusted HR	–	2.13 (1.35–3.38)	1.15 (0.55–2.42)	1.48 (0.64–3.40)	0.791	1.09 (0.99–1.20)
Model 1 HR	–	1.33 (0.79–2.26)	0.70 (0.35–1.41)	0.97 (0.44–2.15)	0.563	1.06 (0.94–1.19)
**Cancer mortality**						
No. of deaths (%)	17 (3.5%)	23 (3.5%)	19 (5.3%)	14 (3.6%)		79 (4.0%)
Unadjusted HR	–	3.34 (1.58–7.05)	2.85 (1.39–5.84)	3.12 (1.79–5.42)	**0.022**	1.09 (1.00–1.19)
Model 1 HR	–	2.51 (1.10–5.71)	2.19 (1.00–4.81)	2.24 (1.10–4.56)	0.270	1.06 (0.93–1.20)

*Model 1: Adjusted for age, race, annual family income, smoking status, and diabetic kidney disease. Model 2: Adjusted for covariates in Model 1 and body mass index, education level, daily carbohydrate consumption, alcohol consumption, years since diabetes diagnosis, diagnosis of hypertension, retinopathy, macrovascular complications, insulin treatment, and survey cycle*.

a *HR for the continuous analysis are presented for each 100 mg increase in caffeine consumption from coffee. HR, Hazard Ratio; CVD, Cardiovascular disease. Significant P for trend values and significant hazard ratios in the continuous analysis are shown in bold*.

Regarding caffeine consumption from tea and from soft drinks, there were no significant associations with all-cause or cause-specific mortality (Supplementary Tables 1,2).

Among men, there were no significant associations between source of caffeine and mortality (Table 3, Supplementary Table 2).

### Sensitivity analysis

The analysis of the association between source of caffeine and mortality using daily intake of caffeine as a continuous variable showed similar results (Table 2, Supplementary Table 3). Among women, the HR for each 100 mg increase in caffeine consumption for all-cause mortality was 0.86 (95% CI, 0.76–0.96; *p* = 0.009) for unadjusted analysis and 0.86 (95% CI, 0.76–0.96; *p* = 0.011) for adjusted analysis. The effect of caffeine consumption according to caffeine source on all-cause, cardiovascular, and cancer mortality among women and men were also concordant with the main analysis (Table 2, Supplementary Table 3). Furthermore, the association of caffeine consumption with mortality adjusting for physical activity was also consistent with our main analysis (Supplementary Table 4).

## Discussion

Our study showed a dose-dependent protective effect of caffeine consumption on all-cause mortality among women with diabetes. There was no significant association between caffeine consumption and mortality among men with diabetes. Although caffeine consumption in women was associated with lower all-cause mortality, no association was found between caffeine consumption and cardiovascular or cancer mortality. When comparing caffeine consumption according to its origin on coffee, tea, or soft drinks, women with diabetes who consumed more caffeine from coffee also had reduced risk of all-cause death. No differences on mortality were observed on the adjusted analysis for consumption of caffeine from tea or soft drinks. However, these results should be interpreted cautiously as the number of events in each source of caffeine category was low.

Previous studies had already shown a protective effect of coffee consumption in the general population. Loftfield et al., for example, showed a decreased risk of all-cause mortality in people with higher coffee consumption (18). Specifically, an inverse association was found between coffee consumption and diabetes-related death. In the subgroup of participants with self-reported diabetes, this association seemed stronger. Regarding studies in people with diabetes, a study in the Finnish population in 3,837 patients, found an inverse relationship between coffee drinking and all-cause and cardiovascular-associated mortality (12). On the other hand, two previous studies have obtained neutral results in patients of both sexes. In a study including only female nurses with diabetes, habitual coffee consumption was not associated with cardiovascular diseases or premature mortality (10). Furthermore, no significant association between coffee consumption and mortality was observed in a prospective cohort of male health professionals with diabetes (11). Although the Finnish population study results are not consistent with these two studies, their results also suggest a protective effect of caffeine-containing beverages as observed in our study. Possible explanations for these differences between studies might be the use of different endpoints, different duration of follow-up and differences in the population's characteristics.

Our results suggest that biological differences may exist between men and women regarding the effects of caffeine consumption. Considering the pathophysiology and complications of diabetes, several studies have highlighted differences between sexes (19). Genetic and epigenetic mechanisms, nutritional factors, and sedentary lifestyle have been shown to differently affect diabetes complications according to sex (19). Furthermore, caffeine may induce different hemodynamic effects in men and women. In a double-blind trial comparing age-matched women and men, women showed an increase in cardiac output, whereas men showed increased vascular resistance after a dietary dose of caffeine (20). These differences may partially explain why caffeine intake is associated with reduced mortality in women with diabetes whereas the effects in mortality are neutral in men with diabetes. In the general population, some studies have also suggested differences in the response to coffee between sexes. The inverse association of coffee drinking with total mortality has been shown to be reduced in men comparing to women (21–23).

The direction and strength of the association between caffeine consumption and mortality has varied between studies in the general population. In a study including Japanese participants without a history of cancer, myocardial infarction, or stroke at baseline, coffee consumption was strongly associated with reduced all-cause and cardiovascular mortality among women [HR of 0.48 (0.29–0.80) for 1–2 cups of coffee per day and 0.45 (0.20–1.03) for 3 or more cups per day comparing with no consumption) but not in men (24). Other studies have also shown inverse associations between consumption of caffeine-containing beverages and mortality among women, albeit with weaker associations (22, 25). Although some studies suggest greater benefits of consumption of caffeine or coffee among women, these findings are not consistent across studies. Several studies have shown similar inverse associations between coffee consumption and mortality in women and men (26–28). The type of caffeine-containing beverage, the population's risk factors or the duration of follow-up may explain the differences between studies.

The benefits of coffee may be directly related to caffeine or to other components present in coffee, including minerals, phytochemicals, and antioxidants (25, 29). The antioxidant capacity of these drinks may contribute to the health-protective effect described with decaffeinated coffee consumption (7). The lack of significant differences on mortality regarding caffeine consumption from tea on our study may be explained by insufficient power. A recent meta-analysis found a significant association between tea consumption and reduction of all-cause mortality; furthermore, black tea was inversely associated with cancer mortality (30).

Tsujimoto et al. also used NHANES data to evaluate the effects of caffeine consumption in the general population (31). In their main analysis, caffeine intake was associated with a decreased risk of all-cause mortality. Although it was not their main objective, the authors also performed an additional analysis limited to the participants with diabetes. Contrary to our results, they reported a non-significant association between caffeine consumption and mortality among participants with diabetes. In the study by Tsujimoto et al., no adjustments were made for diabetes-specific parameters, including diabetes duration, type of treatment and complications of diabetes. Participants with diabetes were not stratified according to sex and no adjustment was performed for the presence of kidney disease. Furthermore, in the study by Tsujimoto et al., participants with missing information on any other potential confounders were excluded, whereas we included these patients using multiple imputation. The differences in participants' selection and in the statistical analyses techniques used probably account for the differences between the studies.

Our study has several strengths, including the evaluation of a cohort of participants from a large database representative of the American population. Data was prospectively collected and included hard outcome measures such as death and cause-specific mortality. The presence of detailed information about the participants allowed for adjustment for the main biologically plausible confounders.

As for limitations, it should be noted that caffeine consumption was evaluated by 24-h dietary recalls. It cannot be excluded that data generated using this method may not represent long-term dietary habits. We consider that the inclusion of data from non-consecutive recalls to estimate usual dietary intake distributions minimizes this risk. Although we present additional information regarding diet in the studied population (such as consumption of carbohydrate, saturated fat, or fiber), no adjustment was performed for additives present in caffeine-containing beverages. Nonetheless, other studies showed significant association between coffee consumption and decreased risk of death even after adjustment for coffee additives, such as cream, milk, sugar, or honey (32). Even though we have found a significant association between caffeine consumption and mortality in women with diabetes, it is possible that the differences found are due to chance, unmeasured confounders, or the possibility that caffeine consumers also perform other protective behaviors, contributing to a healthy user effect. To minimize this possibility, we have considered dietary factors and physical activity as potential confounders. As the number of deaths in our study was low, these estimates should be cautiously interpreted.

In conclusion, this large observational study showed a significant inverse association between caffeine consumption and death from all causes in women with diabetes. These results suggest that advising women with diabetes to drink more caffeine may reduce their mortality. This would represent a simple, clinically beneficial, and inexpensive option in female patients. Further studies, ideally randomized clinical trials, are needed to confirm this benefit. New research should also focus on the different effects of caffeine consumption in men and women and on the benefits of other compounds present in caffeine-containing beverages.

## Data availability statement

The dataset analyzed for this study can be found in: https://www.cdc.gov/nchs/nhanes/index.htm.

## Author contributions

JSN, LL, RM, MBV, and CVD jointly designed and conducted research, developed the analytical strategy and did the statistical analysis. BC reviewed the analytic strategy and statistical analysis. JSN, LL, RM, MBV, and CVD jointly contributed to the first draft. All authors contributed to interpretation of data for the work, critically revised the work, and approved the final version of the manuscript.

### Conflict of interest statement

The authors declare that the research was conducted in the absence of any commercial or financial relationships that could be construed as a potential conflict of interest.
