# Supplementary material for: Caffeine Consumption and Mortality in Diabetes: An Analysis of NHANES 1999–2010
Source: Front Endocrinol (Lausanne). 2018 Sep 20;9:547. doi: 10.3389/fendo.2018.00547 (PMC6158371; doi:10.3389/fendo.2018.00547)
Supplement: Supplementary file 1 [file Table_1.DOCX]

| **Supplementary Table 1 - Association of source of caffeine consumption (tea or soft drinks) with mortality among women** | | | | | |
| --- | --- | --- | --- | --- | --- |
| **Caffeine from Tea** | **No consumption  (n=1412)** | **Low tertile  (n=196)** | **Middle tertile  (n=197)** | **High tertile  (n=169)** | **P for trend** |
| **All-cause mortality** |  |  |  |  |  |
| No. of deaths (%) | 264 (18.7%) | 29 (14.8%) | 32 (16.2%) | 26 (15.4%) |  |
| Unadjusted HR | - | 0.74 (0.46-1.18) | 1.01 (0.65-1.57) | 0.81 (0.48-1.38) | 0.492 |
| Model 1 HR | - | 0.62 (0.38-1.01) | 0.82 (0.55-1.22) | 0.99 (0.58-1.69) | 0.888 |
| Model 2 HR | - | 0.57 (0.35-0.92) | 0.86 (0.60-1.23) | 0.96 (0.57-1.61) | 0.860 |
| **CVD mortality** |  |  |  |  |  |
| No. of deaths (%) | 59 (4.2%) | 4 (4.2%) | 11 (5.6%) | 5 (3.0%) |  |
| Unadjusted HR | - | 0.32 (0.09-1.12) | 1.85 (0.94-3.65) | 0.46 (0.14-1.59) | 0.371 |
| Model 1 HR | - | 0.24 (0.06-0.91) | 1.39 (0.68-2.82) | 0.57 (0.16-2.08) | 0.595 |
| **Cancer mortality** |  |  |  |  |  |
| No. of deaths (%) | 42 (3.0%) | 6 (3.1%) | 2 (1.0%) | 4 (2.4%) |  |
| Unadjusted HR | - | 0.92 (0.35-2.39) | 0.22 (0.05-1.01) | 0.50 (0.14-1.82) | 0.222 |
| Model 1 HR | - | 0.91 (0.33-2.50) | 0.21 (0.04-0.98) | 0.60 (0.16-2.24) | 0.309 |
| **Caffeine from   Soft Drinks** | **No consumption  (n=1333)** | **Low tertile  (n=278)** | **Middle tertile  (n=201)** | **High tertile  (n=162)** | **P for trend** |
| **All-cause mortality** |  |  |  |  |  |
| No. of deaths (%) | 277 (20.8%) | 36 (13.0%) | 23 (11.4%) | 15 (9.3%) |  |
| Unadjusted HR | - | 0.60 (0.38-0.94) | 0.36 (0.20-0.64) | 0.37 (0.19-0.72) | **<0.001** |
| Model 1 HR | - | 0.64 (0.40-1.03) | 0.57 (0.31-1.04) | 0.64 (0.31-1.31) | 0.093 |
| Model 2 HR | - | 0.65 (0.39-1.11) | 0.60 (0.33-1.09) | 0.66 (0.33-1.32) | 0.117 |
| **CVD mortality** |  |  |  |  |  |
| No. of deaths (%) | 59 (4.4%) | 12 (4.3%) | 4 (2.0%) | 4 (2.5%) |  |
| Unadjusted HR | - | 0.95 (0.45-2.02) | 0.35 (0.11-1.15) | 0.45 (0.13-1.60) | 0.126 |
| Model 1 HR | - | 1.03 (0.47-2.27) | 0.60 (0.17-1.87) | 0.84 (0.23-3.05) | 0.636 |
| **Cancer mortality** |  |  |  |  |  |
| No. of deaths (%) | 41 (3.1%) | 3 (3.2%) | 3 (1.5%) | 1 (0.6%) |  |
| Unadjusted HR | - | 1.01 (0.40-2.58) | 0.07 (0.02-0.22) | 0.21 (0.03-1.46) | 0.055 |
| Model 1 HR | - | 0.98 (0.37-2.61) | 0.09 (0.03-0.29) | 0.30 (0.04-1.98) | 0.120 |

Supplementary Table 1. Association of caffeine consumption from tea or soft drinks with all-cause, cardiovascular disease, and cancer-specific mortality among women. Model 1: Adjusted for age, race, annual family income, smoking status, and diabetic kidney disease. Model 2: Adjusted for covariates in Model 1 and body mass index, education level, daily carbohydrate consumption, alcohol consumption, years since diabetes diagnosis, diagnosis of hypertension, retinopathy, macrovascular complications, insulin treatment and survey cycle. HR: Hazard Ratio, CVD: Cardiovascular disease.
