# Supplementary material for: Caffeine Consumption and Mortality in Diabetes: An Analysis of NHANES 1999–2010
Source: Front Endocrinol (Lausanne). 2018 Sep 20;9:547. doi: 10.3389/fendo.2018.00547 (PMC6158371; doi:10.3389/fendo.2018.00547)
Supplement: Supplementary file 2 [file Table_2.DOCX]

| **Supplementary Table 2 - Association of source of caffeine consumption (tea or soft drinks) with mortality among men** | | | | | |
| --- | --- | --- | --- | --- | --- |
| **Caffeine from Tea** | **No consumption  (n=1440)** | **Low tertile  (n=206)** | **Middle tertile  (n=185)** | **High tertile  (n=143)** | **P for trend** |
| **All-cause mortality** |  |  |  |  |  |
| No. of deaths (%) | 315 (21.9%) | 36 (17.5%) | 33 (17.8%) | 23 (16.1%) |  |
| Unadjusted HR | - | 0.77 (0.48-1.23) | 0.78 (0.46-1.31) | 0.75 (0.40-1.44) | 0.307 |
| Model 1 HR | - | 0.70 (0.43-1.12) | 0.67 (0.39-1.13) | 0.86 (0.48-1.53) | 0.348 |
| Model 2 HR | - | 0.66 (0.39-1.09) | 0.67 (0.40-1.10) | 0.84 (0.49-1.44) | 0.274 |
| **CVD mortality** |  |  |  |  |  |
| No. of deaths (%) | 95 (6.6%) | 10 (4.9%) | 7 (3.8%) | 8 (5.6%) |  |
| Unadjusted HR | - | 0.82 (0.38-1.75) | 0.35 (0.12-1.00) | 0.88 (0.36-2.19) | 0.526 |
| Model 1 HR | - | 0.76 (0.38-1.52) | 0.28 (0.10-0.80) | 1.05 (0.45-2.45) | 0.601 |
| **Cancer mortality** |  |  |  |  |  |
| No. of deaths (%) | 60 (4.2%) | 6 (2.9%) | 8 (4.3%) | 5 (3.5%) |  |
| Unadjusted HR | - | 0.76 (0.25-2.29) | 1.55 (0.59-4.08) | 1.26 (0.37-4.30) | 0.525 |
| Model 1 HR | - | 0.76 (0.24-2.42) | 1.33 (0.51-3.49) | 1.23 (0.37-4.06) | 0.594 |
| **Caffeine from   Soft Drinks** | **No consumption  (n=1231)** | **Low tertile  (n=338)** | **Middle tertile  (n=237)** | **High tertile  (n=168)** | **P for trend** |
| **All-cause mortality** |  |  |  |  |  |
| No. of deaths (%) | 290 (23.6%) | 51 (15.1%) | 36 (15.2%) | 30 (17.9%) |  |
| Unadjusted HR | - | 0.88 (0.60-1.30) | 0.55 (0.37-0.80) | 0.78 (0.48-1.27) | 0.217 |
| Model 1 HR | - | 0.94 (0.65-1.35) | 0.64 (0.42-1.00) | 1.17 (0.70-1.95) | 0.777 |
| Model 2 HR | - | 1.00 (0.71-1.41) | 0.63 (0.39-1.02) | 1.08 (0.67-1.74) | 0.953 |
| **CVD mortality** |  |  |  |  |  |
| No. of deaths (%) | 82 (6.7%) | 14 (4.1%) | 12 (5.1%) | 12 (7.1%) |  |
| Unadjusted HR | - | 0.97 (0.50-1.88) | 0.69 (0.34-1.40) | 0.81 (0.35-1.90) | 0.563 |
| Model 1 HR | - | 1.02 (0.54-1.94) | 0.78 (0.39-1.57) | 1.28 (0.52-3.20) | 0.700 |
| **Cancer mortality** |  |  |  |  |  |
| No. of deaths (%) | 54 (4.4%) | 12 (3.6%) | 9 (3.8%) | 4 (2.4%) |  |
| Unadjusted HR | - | 1.00 (0.36-2.76) | 0.61 (0.25-1.49) | 0.61 (0.18-2.07) | 0.353 |
| Model 1 HR | - | 1.03 (0.38-2.83) | 0.70 (0.27-1.82) | 0.82 (0.25-2.77) | 0.667 |

Supplementary Table 2. Association of caffeine consumption from tea or soft drinks with all-cause, cardiovascular disease, and cancer-specific mortality among men. Model 1: Adjusted for age, race, annual family income, smoking status, and diabetic kidney disease. Model 2: Adjusted for covariates in Model 1 and body mass index, education level, daily carbohydrate consumption, alcohol consumption, years since diabetes diagnosis, diagnosis of hypertension, retinopathy, macrovascular complications, insulin treatment and survey cycle. HR: Hazard Ratio, CVD: Cardiovascular disease.
