# Supplementary material for: Caffeine Consumption and Mortality in Diabetes: An Analysis of NHANES 1999–2010
Source: Front Endocrinol (Lausanne). 2018 Sep 20;9:547. doi: 10.3389/fendo.2018.00547 (PMC6158371; doi:10.3389/fendo.2018.00547)
Supplement: Supplementary file 3 [file Table_3.docx]

| **Supplementary Table 3 -** **Association of caffeine consumption from tea or soft drinks (as a continuous variable) with mortality among women and men** | | | | |
| --- | --- | --- | --- | --- |
|  | **Women** | | **Men** | |
|  | **Hazard Ratio** | **P value** | **Hazard Ratio** | **P value** |
| **Caffeine from tea** |  |  |  |  |
| **All-cause mortality** |  |  |  |  |
| Unadjusted HR | 0.83 (0.65-1.07) | 0.150 | 0.93 (0.73-1.17) | 0.520 |
| Model 1 HR | 0.93 (0.71-1.21) | 0.570 | 0.93 (0.78-1.26) | 0.945 |
| Model 2 HR | 0.92 (0.72-1.17) | 0.489 | 1.01 (0.80-1.29) | 0.921 |
| **CVD mortality** |  |  |  |  |
| Unadjusted HR | 0.87 (0.55-1.37) | 0.540 | 0.89 (0.59-1.33) | 0.561 |
| Model 1 HR | 0.98 (0.59-1.62) | 0.925 | 0.94 (0.58-1.52) | 0.800 |
| **Cancer mortality** |  |  |  |  |
| Unadjusted HR | 0.68 (0.27-1.76) | 0.425 | 1.19 (0.92-1.53) | 0.187 |
| Model 1 HR | 0.75 (0.28-1.97) | 0.550 | 1.25 (0.95-1.64) | 0.107 |
| **Caffeine from soft drinks** |  |  |  |  |
| **Death from all causes** |  |  |  |  |
| Unadjusted HR | **0.47 (0.22-0.99)** | **0.047** | 0.93 (0.66-1.32) | 0.689 |
| Model 1 HR | 0.79 (0.44-1.41) | 0.416 | 1.16 (0.88-1.52) | 0.289 |
| Model 2 HR | 0.78 (0.46-1.33) | 0.364 | 1.03 (0.82-1.31) | 0.772 |
| **CVD mortality** |  |  |  |  |
| Unadjusted HR | 0.62 (0.19-2.03) | 0.427 | 1.05 (0.59-1.88) | 0.869 |
| Model 1 HR | 1.03 (0.40-2.60) | 0.955 | 1.29 (0.86-1.93) | 0.220 |
| **Cancer mortality** |  |  |  |  |
| Unadjusted HR | 0.22 (0.03-1.60) | 0.134 | 0.57 (0.30-1.10) | 0.093 |
| Model 1 HR | 0.34 (0.05-2.11) | 0.243 | 0.70 (0.35-1.39) | 0.300 |

Supplementary Table 3. Association of total caffeine consumption and caffeine consumption from tea or soft drinks with all-cause, cardiovascular disease, and cancer-specific mortality among women and men. HR for each 100 mg increase in caffeine consumption. Model 1: Adjusted for age, race, annual family income, smoking status, and diabetic kidney disease. Model 2: Adjusted for Model 1 and body mass index, education level, daily carbohydrate consumption, alcohol consumption, years since diabetes diagnosis, diagnosis of hypertension, retinopathy, macrovascular complications, insulin treatment and survey cycle. HR: Hazard Ratio, CVD: Cardiovascular disease.
