# Supplementary material for: Caffeine Consumption and Mortality in Diabetes: An Analysis of NHANES 1999–2010
Source: Front Endocrinol (Lausanne). 2018 Sep 20;9:547. doi: 10.3389/fendo.2018.00547 (PMC6158371; doi:10.3389/fendo.2018.00547)
Supplement: Supplementary file 4 [file Table_4.docx]

| **Supplementary Table 4 - Association of total caffeine consumption and caffeine consumption from coffee with total mortality adjusted for physical activity** | | | | | |
| --- | --- | --- | --- | --- | --- |
| **Association of total caffeine consumption and caffeine consumption from coffee with total mortality among women adjusted for physical activity** | | | | | |
|  | **No consumption** | **<100 mg/day** | **100 to <200 mg/day** | **≥200 mg/day** | **P for trend** |
| **Total caffeine** |  |  |  |  |  |
| No. of deaths (%) | 59 (26.9%) | 170 (17.4%) | 73 (16.7%) | 49 (14.5%) |  |
| Unadjusted HR | - | 0.71 (0.51-1.00) | 0.63 (0.42-0.96) | 0.46 (0.27-0.78) | **0.010** |
| Model 3 | - | 0.60 (0.42-0.85) | 0.54 (0.35-0.83) | 0.40 (0.24-0.67) | **0.008** |
| **Caffeine from coffee** |  |  |  |  |  |
| No. of deaths (%) | 127 (17.3%) | 130 (18.4%) | 58 (18.4%) | 36 (16.6%) |  |
| Unadjusted HR | - | 1.25 (0.94-1.66) | 1.09 (0.75-1.60) | 0.73 (0.50-1.09) | 0.077 |
| Model 3 | - | 0.80 (0.58-1.09) | 0.80 (0.51-1.23) | 0.58 (0.39-0.86) | **0.012** |
| **Association of total caffeine consumption and caffeine consumption from coffee with total mortality among men adjusted for physical activity** | | | | | |
|  | **No consumption** | **<100 mg/day** | **100 to <200 mg/day** | **≥200 mg/day** | **P for trend** |
| **Total caffeine** |  |  |  |  |  |
| No. of deaths (%) | 50 (26.9%) | 159 (21.5%) | 82 (17.5%) | 116 (20.0%) |  |
| Unadjusted | - | 1.24 (0.81-1.91) | 1.00 (0.64-1.57) | 1.21 (0.81-1.82) | 0.739 |
| Model 3 | - | 1.09 (0.70-1.67) | 0.92 (0.58-1.47) | 1.01 (0.65-1.58) | 0.826 |
| **Caffeine from coffee** |  |  |  |  |  |
| No. of deaths (%) | 132 (20.1%) | 126 (22.0%) | 70 (19.6%) | 79 (20.4%) |  |
| Unadjusted HR | - | 1.59 (1.19-2.13) | 1.13 (0.77-1.66) | 1.42 (0.97-2.08) | 0.282 |
| Model 3 | - | 1.07 (0.77-1.49) | 0.82 (0.59-1.15) | 1.14 (0.80-1.64) | 0.668 |

Supplementary Table 4. Association of total caffeine consumption and caffeine consumption from coffee with total mortality adjusting for physical activity. Model 3: Adjusted for age, race, annual family income, smoking status and diabetic kidney disease, body mass index, education level, daily carbohydrate consumption, alcohol consumption, years since diabetes diagnosis, diagnosis of hypertension, retinopathy, macrovascular complications, insulin treatment, survey cycle and physical activity level. HR: Hazard Ratio.
